# Causality of genetically determined metabolites and metabolic pathways on osteoarthritis: a two-sample mendelian randomization study

**DOI:** 10.1186/s12967-023-04165-9

**Published:** 2023-05-31

**Authors:** Yifei Gu, Qianmei Jin, Jinquan Hu, Xinwei Wang, Wenchao Yu, Zhanchao Wang, Chen Wang, Yang Liu, Yu Chen, Wen Yuan

**Affiliations:** 1 Department of Orthopaedics, Changzheng Hospital, Naval Medical University, Shanghai, 200003 China; 2 Department of Rheumatology and Immunology, Changzheng Hospital, Naval Medical University, Shanghai, 200003 China

**Keywords:** Osteoarthritis, Genetically determined metabolites, Mendelian randomization, Arginine, Kynurenine

## Abstract

**Background:**

Osteoarthritis (OA) is one of the most prevalent musculoskeletal diseases and is the leading cause of pain and disability in the aged population. However, the underlying biological mechanism has not been fully understood. This study aims to reveal the causal effect of circulation metabolites on OA susceptibility.

**Methods:**

A two-sample Mendelian Randomization (MR) analysis was performed to estimate the causality of GDMs on OA. A genome-wide association study (GWAS) of 486 metabolites was used as the exposure, whereas 8 different OA phenotypes, including any-site OA (All OA), knee and/or hip OA (knee/hip OA), knee OA, hip OA, spine OA, finger and/or thumb OA (hand OA), finger OA, thumb OA, were set the outcomes. Inverse-variance weighted (IVW) was used for calculating causal estimates. Methods including weight mode, weight median, MR-egger, and MR-PRESSO were used for the sensitive analysis. Furthermore, metabolic pathway analysis was performed via the web-based Metaconflict 4.0. All statistical analyses were performed in R software.

**Results:**

In this MR analysis, a total of 235 causative associations between metabolites and different OA phenotypes were observed. After false discovery rate (FDR) correction and sensitive analysis, 9 robust causative associations between 7 metabolites (e.g., arginine, kynurenine, and isovalerylcarnitine) and 5 OA phenotypes were finally identified. Additionally, eleven significant metabolic pathways in 4 OA phenotypes were identified by metabolic pathway analysis.

**Conclusion:**

The finding of our study suggested that identified metabolites and metabolic pathways can be considered useful circulating metabolic biomarkers for OA screening and prevention in clinical practice, and can also serve as candidate molecules for future mechanism exploration and drug target selection.

**Supplementary Information:**

The online version contains supplementary material available at 10.1186/s12967-023-04165-9.

## Introduction

Osteoarthritis (OA) is the most prevalent degenerative musculoskeletal disorder and is the leading cause of progressive pain and chronic disability in the aged population. OA can affect various synovial joints, with knee joints most commonly involved, followed by the lumbar spine, cervical spine, hands, ankle, and hip joints [1]. With a growing global population and increasing life expectancy, OA has become a major public health problem due to its high prevalence and disability rate. From the recent Global Burden of Disease estimation [2], approximately 344 million people worldwide suffer from osteoarthritis, with a 114% increase in prevalence and a 115% increase in the population living with disability since 1990. Although the pathological process of OA formation varies in different types of joints, it is generally characterized by progressive degradation of the articular cartilage along with secondary episodic synovitis and bone remodeling [3]. Although considerable efforts have been undertaken to understand the nature of OA, the mechanism and risk factors of OA are still elusive.

In recent years, there are growing evidence that metabolic dysregulation has a close association with the development of OA. Both in vivo and in vitro studies indicated potential links between OA and metabolic disorders including hypertension, diabetes mellitus (or insulin resistance), dyslipidemia, and obesity [4–8]. There are also studies suggesting that they may share common pathological processes or pathways [4, 5]. Currently, OMIC technologies, including genomics and metabolomics, have been introduced into the investigation of underlying pathophysiological mechanisms and potential therapeutic strategies for human diseases. Several recent metabolomic studies have reported numerous circulating biomarkers including amino acids, carbohydrates, and lipids in both human and animal models [9–12]. However, due to sample size limitations and confounding factors, the causal effect of blood metabolites on OA still cannot be confirmed.

With the development of high-throughput technologies, measuring hundreds of circulating metabolites and performing genotyping in large-scale populations in parallel are now capable [13]. Genome-wide association studies (GWAS) can offer molecular insights into the complex interplay between environmental and genetic factors in the pathogenesis of diseases. Additionally, a considerable number of single nucleotide polymorphisms (SNPs) have been identified with strong associations with serum metabolites. However, there is still a great barrier to translating these genetic findings into biological mechanisms of OA development, which calls for deep analysis to reveal the causal interaction of serum metabolites on OA susceptibility.

Mendelian randomization (MR) analysis is a novel and powerful epidemiological tool that uses genetic variants as unconfounded instrumental variables to investigate the causal relationships among exposures and clinical outcomes of diseases. Exploiting the fact that genotypes are determined at conception and not generally susceptible to confounders, MR analysis can provide unbiased estimates. Recently, GWAS were extended to metabolic phenotypes which generated an atlas of genetically determined metabolites (GDMs) [14]. Inspired by the given dataset of GDMs, we hereby employed systematic MR methods to (1) assess the causal effects of human serum metabolites on OA in both weight-bearing and non-weight-bearing joints; (2) identify common metabolites that had causal effects on multiple OA phenotype; and (3) identify metabolic pathways that might contribute to the development of the OA.

## Material and methods

### Study design

**Fig. 1:**
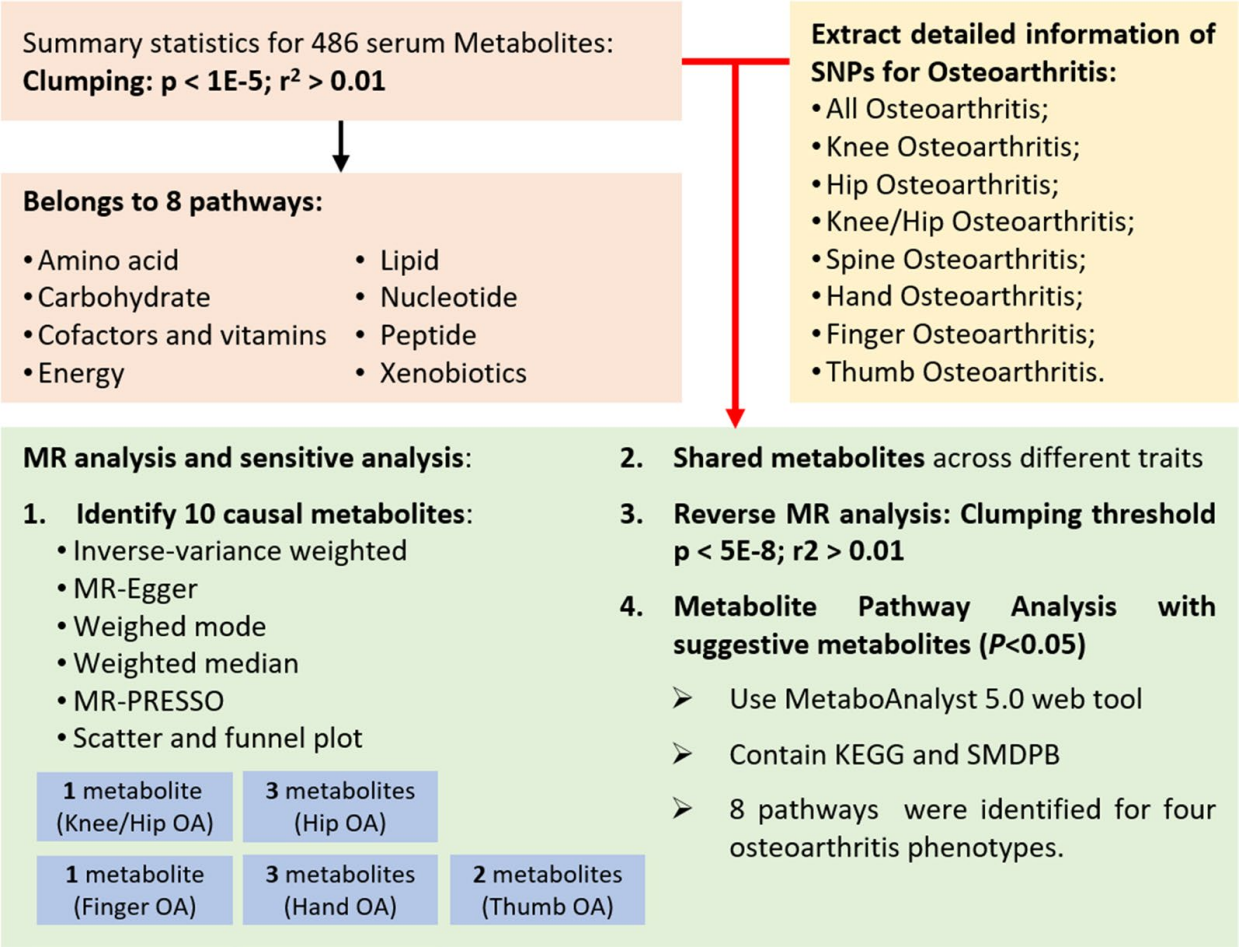
The overview of the research workflow

We systematically assessed the causal association between human circulating metabolites and the risk of OA using a two-sample MR design. The performance of a convincing MR study should comply with 3 fundamental assumptions: (1) the genetic instruments are supposed to have direct associations with exposure (i.e., metabolites in this study); (2) the genetic instruments are supposed to be unrelated with the outcome (i.e., OA in this study) and independent of any known or unknown confounding factors; (3) the effects of IVs on the outcomes are solely mediated by the exposures of interest. Genetic information for metabolites and osteoarthritis was obtained from independent GWAS datasets separately to avoid sample overlap. The overview of this MR study was presented in Fig. 1.

### GWAS data for human serum metabolites

The genome-wide association summary datasets involving 486 metabolites were obtained from the study by Shin et al. [14]. This is the currently most comprehensive analysis of human metabolites, and the full summary statistics of which were publicly available via Metabolomics GWAS Server (http://metabolomics.helmholtz-muenchen.de/gwas/). A total of 7824 adult individuals from two European cohorts (TwinsUK and KORA cohorts) and approximately 2.1 million SNPs were included in this GWAS analysis. Among the 486 metabolites, 309 were known metabolites that can be assigned to 8 broad metabolic groups (amino acids, carbohydrates, cofactors and vitamins, energy, lipids, nucleotides, peptides, and xenobiotic metabolism) as defined in the Kyoto Encyclopedia of Genes and Genomes (KEGG) database[15] (Additional file 1: Table S2). Another 177 were unknown metabolites whose chemical identity had not been conclusively determined.

### GWAS data for OA

The summary datasets of OA were derived from a GWAS meta-analysis of 826,690 individuals (177517 OA patients and 649173 controls) from 9 different populations, this study identified 100 independently associated risk variants in 11 osteoarthritis phenotypes and is the most comprehensive GWAS analysis available on osteoarthritis [16]. After excluding surgical phenotypes that were not relevant to the purpose of this study, we selected 8 OA phenotypes for further analysis, which were any-site OA (All OA), knee and/or hip OA (knee/hip OA), knee OA, hip OA, spine OA, finger and/or thumb OA (hand OA), finger OA, thumb OA. The characteristics of the summary datasets for OA were shown in Table 1 The definition of OA in the datasets included self-reported OA, clinical diagnosis, and the tenth edition of the International Classification of Diseases (ICD-10). According to the function of these OA-involved joints, they can be divided into weight-bearing joints (knee, hip, and spine) and non-weight-bearing joints (finger, thumb). Useful information for each SNP (e.g., effect size, standard error, effect allele, and P value) was retained for further analysis.

**Table 1:** Characteristics of the Summary Datasets for Osteoarthritis

OA phenotypes	Sample size	Case	Control	Prevalence (%)
All OA	826690	177,517	649173	21.47
Knee/Hip OA	490345	89,741	400604	18.30
Hip OA	353388	36,445	316943	10.31
Knee OA	396054	62,497	333557	15.78
Hand OA	303782	20,901	282881	6.88
Finger OA	266618	10,804	255814	4.05
Thumb OA	247455	10,536	236919	4.26
Spine OA	333950	28,372	305578	8.50

To ensure the rationality of our analysis, we performed quality control of SNPs by removing non-dual allele SNPs, all SNPs with strand ambiguous alleles, SNPs without rsID, SNPs with duplicate rsID or base pair positions, SNPs not in the 1000 Genomes Project phase 3, SNPs with base pair positions or alleles that do not match those in 1000 Genomes Project phase 3, SNPs with interpolation information < 0.9 and all SNPs on chromosome X and Y.

### Selection of instrumental variables

The selection of instrumental variables (IV) in this MR analysis was based on the 3 fundamental assumptions. Firstly, for each metabolite, we extracted SNPs with association thresholds at P < 1 × 10^–5^. Secondly, independent variants were identified using a clumping procedure implemented in R software, in which a linkage-disequilibrium threshold of R^2^ < 0.001 within a 500 kilobase (kb) distance in the European 1000 Genomes Project Phase 3 reference panel was set. Finally, to quantitatively verify whether the selected SNPs were strong instruments, we calculated the proportion of phenotypic variation explained (PVE) and the *F* statistic of instruments for each metabolite. Typically, a threshold of *F* > 10 was suggested for further MR analysis.

### MR analysis

The causal associations between metabolites and OA for this MR analysis were mainly estimated using a standard inverse variance weighted (IVW) method. When the instrumental variables satisfy all 3 assumptions, the IVW method can provide consistent estimates of the causal effect of the exposure and is considered to be the strongest MR method. However, if some instruments contradict the IV assumptions, the analysis may give incorrect results. We have therefore performed the following sensitivity analysis: (1) Q-test for IVW and MR-Egger was used to detect potential violations of the assumption by the heterogeneity of the association between individual IVs; (2) MR-Egger was applied to estimate horizontal pleiotropy according to its intercept, ensuring that genetic variation was independently associated with exposure and outcome; (3) we applied the additional analyses of MR methods with different modeling assumptions and strengths (weighted median and weighted mode) to increase the stability and robustness of the results; (4) we applied MR-PRESSO to detect outliers and correct for horizontal pleiotropy; (5) we applied individual SNP analysis and leave-one-out analysis to assess the likelihood of associations observed by individual SNP driver.

### Metabolic pathway analysis

Metabolic pathways were analyzed via the web-based Metaconflict 4.0. (https://www.metaboanalyst.ca/) [17]. The functional enrichment analyses and pathway analyses module were used to identify potential metabolite groups or pathways that may be relevant to the biological processes of OA. Two libraries, the Small Molecule Pathway Database (SMPDB) and the Kyoto Encyclopedia of Genes and Genomes (KEGG) database were used in this study, and the significance level for pathway analysis was set at 0.10.

### Statistical analysis

Statistical analyses were performed in R3.5.3 software, and MR analyses were performed using the MendelianRandomization package. MR-PRESSO was performed using the MRPRESSO package. False discovery rate (FDR) correction was used to control for false positives in multiple testing. A statistically significant association was considered if the estimated causal effect of a given metabolite had a FDR < 0.05.

## Results

### Selection of IVs

The number of selected IVs for 486 metabolites ranged from 3 to 631, with a median number of 15 (Additional file 1: Table S4). These generated IVs could explain 0.0156–3.327% of the variance of their respective metabotypes. Importantly, the minimum *F* statistics for the validity test were all above 10 (ranging from 17.63 to 21.96) (Additional file 1: Table S4), indicating that weak instrumental bias is unlikely to occur [18].

### Causal effects of metabolites on 8 OA phenotypes

**Fig. 2:**
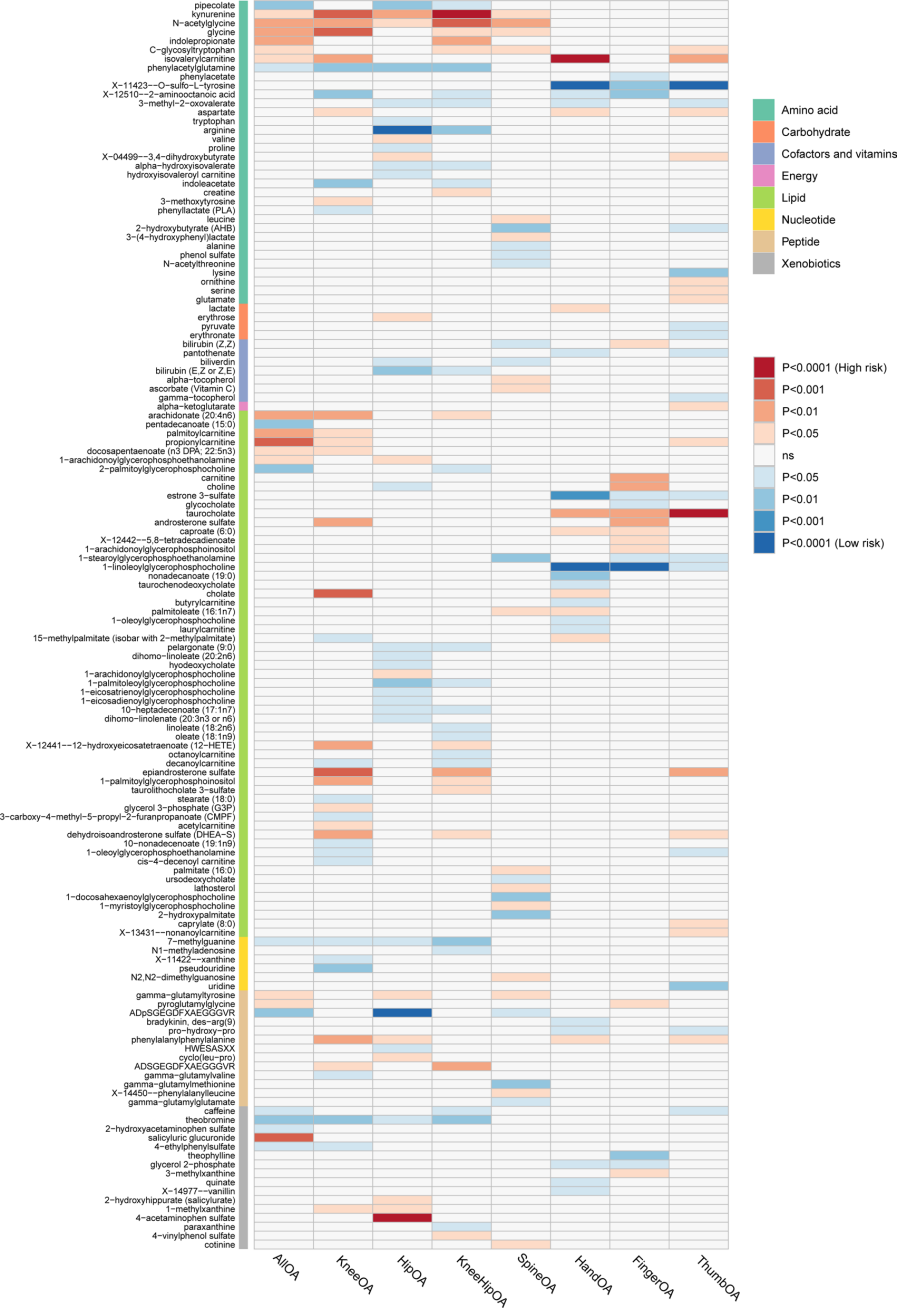
Mendelian randomization associations of known metabolites on the risk of the 8 phenotypes of osteoarthritis. (derived from the fixed-effect IVW analysis). *IVW* inverse-variance weighted)

**Fig. 3:**
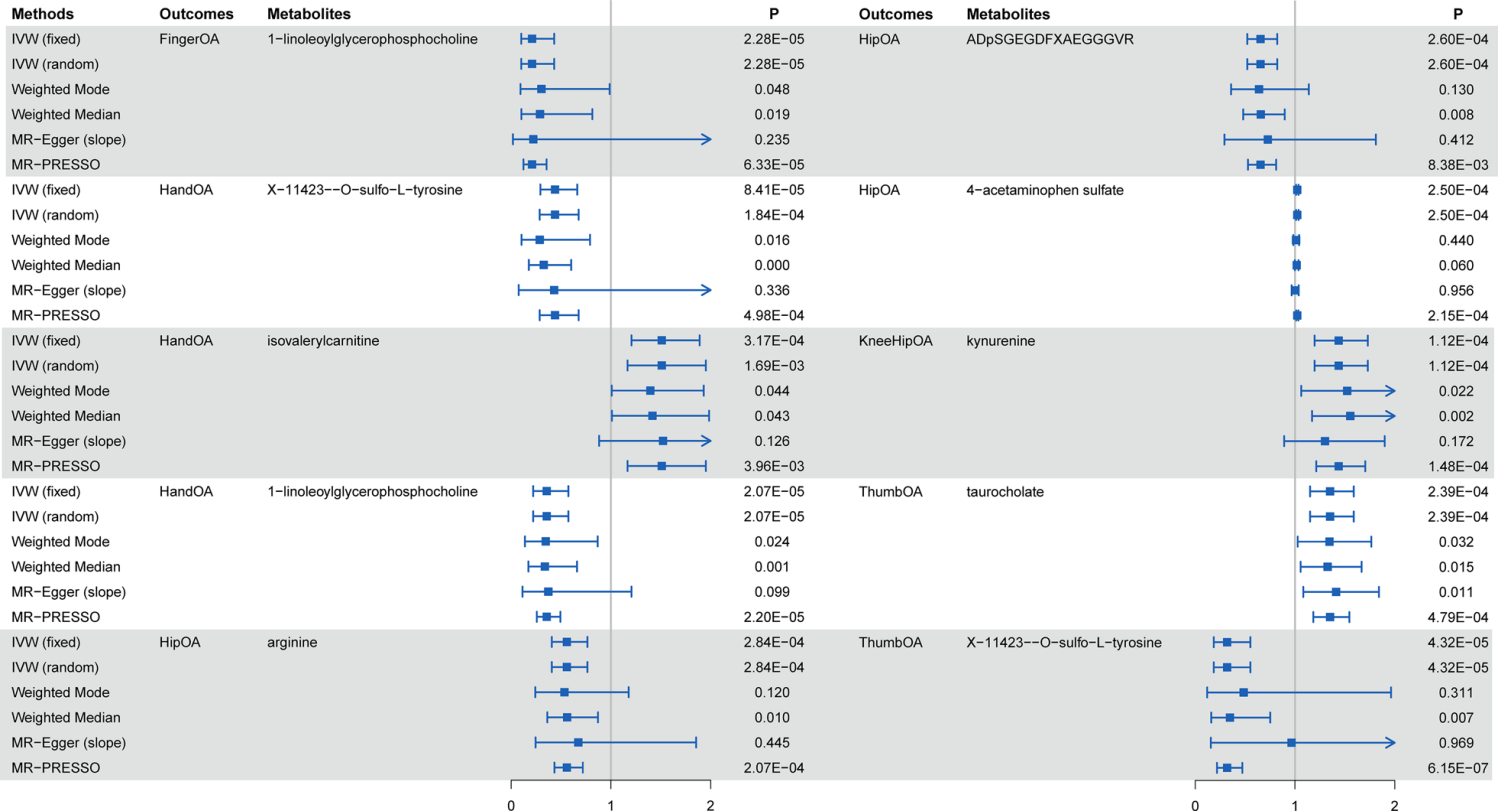
Sensitivity analysis for significant metabolites on OA phenotypes passing Bonferroni correction

For a better interpretation of metabolic changes, we excluded 177 unknown metabolites while including 309 with known structures and functions. Using these IVs, we estimated the causal association between these 309 metabolites and 8 OA phenotypes and identified a total of 235 suggestive associations (p < 0.05, corresponding to 140 unique metabolites)** (**Fig. 2**)**. Using the IVW method, 10 causal associations with multiple-testing corrected significance (FDR < 0.05) could be observed, involving eight metabolites, which included four metabolites from the amino acid pathways, two from the lipid metabolism pathways, one from the peptide pathways and one from the xenobiotic pathways **(**Fig. 3**)**. They were as follows: finger OA, 1-linoleoylglycerophosphocholine (odds ratio [OR] = 0.210, 95% confidence intervals CI 0.102–0.432, FDR < 0.001, p = 2.28 × 10^–5^); hand OA, X-11423–O-sulfo-L-tyrosine (OR = 0.440, 95% CI 0.293–0.663, FDR < 0.001, p = 8.41 × 10^–5^); hand OA, isovalerylcarnitine (OR = 1.510,95% CI 0.293–0.663,FDR < 0.001, p = 3.17 × 10^–4^); hand OA, 1-linoleoylglycerophosphocholine (OR = 0.357, 95% CI 0.222–0.574, FDR < 0.001, p = 2.07 × 10^–5^); hip OA, arginine (OR = 0.559, 95% CI 0.408–0.765, FDR < 0.001, p = 2.84 × 10^–4^); hip OA, ADpSGEGDFXAEGGGVR* (OR = 0.655, 95% CI 0.552–0.822, FDR < 0.001, p = 2.60 × 10^–4^); hip OA, 4-acetaminophen sulfate (OR = 1.023, 95% CI 1.011 = 1.036, FDR < 0.001, p = 2.50 × 10^–4^); knee-hip OA, kynurenine (OR = 1.438, 95% CI 1.196–1.730, FDR < 0.001, p = 1.12 × 10^–4^); thumb OA, taurocholate (OR = 1.352, 95% CI 1.151–1.589, FDR < 0.001, p = 2.39 × 10^–4^); thumb OA, X-11423–O-sulfo-L-tyrosine (OR = 0.320, 95% CI 0.186–0.553, FDR < 0.001, p = 4.32 × 10^–5^).

Additionally, we observed that some OA phenotypes have shared causal metabolites. For example, 1-linoleoylglycerophosphocholine had significant causal associations with both finger OA and hand OA, as well as a suggestive causal association with thumb OA (OR = 0.4398, 95% CI 0.221–0.873, p = 0.01889). X-11423-O-sulfo-L-tyrosine had causal associations with both hand OA, thumb OA, and finger OA (OR = 0.400, 95% CI 0.223–0.718, p = 0.00213). Besides knee-hip OA, kynurenine had been found to have suggestive associations with knee OA (OR = 1.435, 95% CI 1.150–1.789, p = 0.00133), hip OA (OR = 1.450, 95% CI 1.082–2.064, p = 0.00699), all OA (OR = 1.194, 95% CI 1.037–1.376, p = 0.01368) and spine OA (OR = 1.494, 95% CI 1.083–2.064, p = 0.01465). Interestingly, the associations between shared metabolites with OA phenotypes showed a trend in the differential distribution between weight-bearing and non-weight-bearing joints.

### Sensitive analysis

**Fig. 4:**
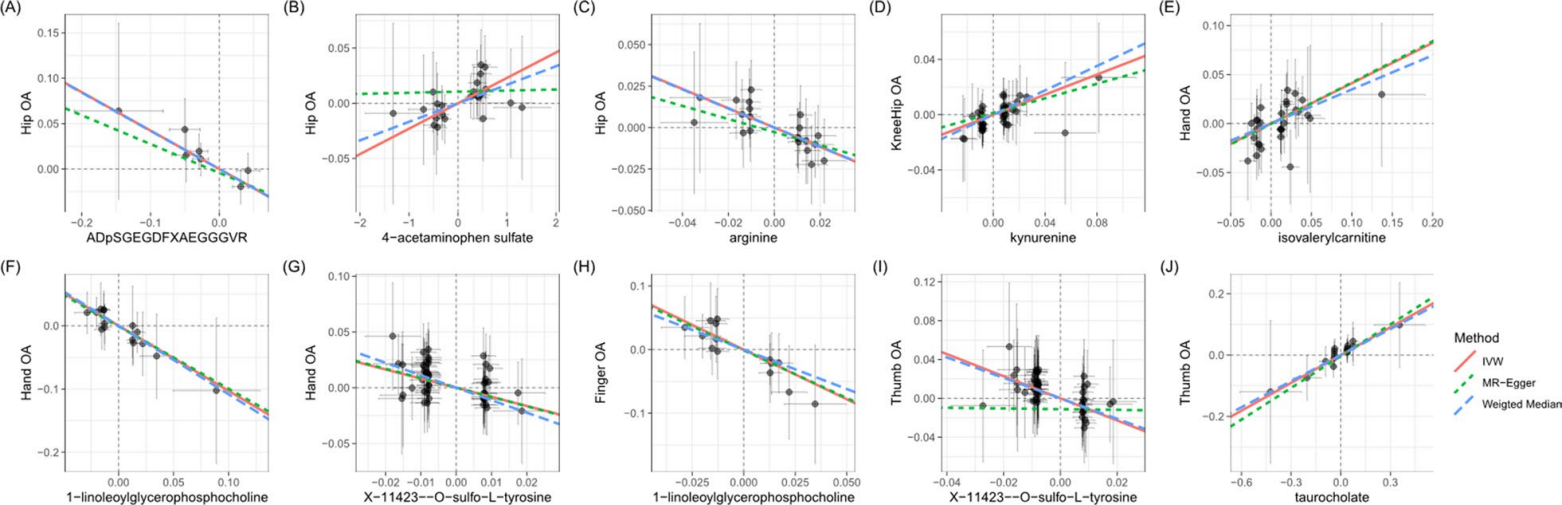
Scatter plot showing the genetic associations of seven metabolites on the risk of 5 OA phenotypes. (**A**) ADpSGEGDFXAEGGGVR* on hip OA, (**B**) 4-acetaminophen sulfate on hip OA, (**C**) arginine on hip OA, (**D**) kynurenine on knee/hip OA, (**E**) isovalerylcarnitine on hand OA, (**F**) 1-linoleoylglycerophosphocholine on hand OA, (**G**) X-11423–O-sulfo-L-tyrosine on hand OA, (**H**) 1-linoleoylglycerophosphocholine on finger OA, (**I**) X-11423–O-sulfo-L-tyrosine on thumb OA, (**J**) taurocholate on thumb OA *OA* osteoarthritis

**Fig. 5:**
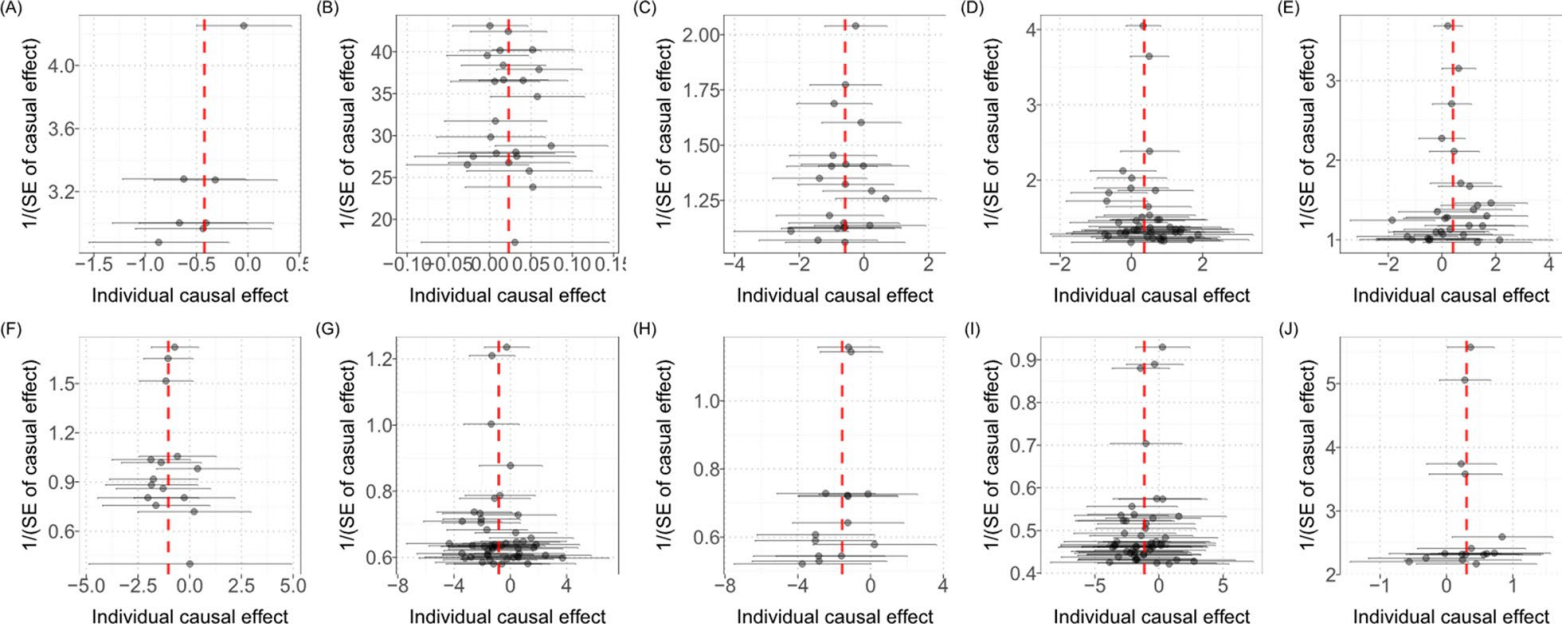
The funnel plot represents IVs for each significant causal association between metabolites and OA phenotypes. **A** ADpSGEGDFXAEGGGVR* on hip OA, (**B**) 4-acetaminophen sulfate on hip OA, (**C**) arginine on hip OA, (**D**) kynurenine on knee/hip OA, (**E**) isovalerylcarnitine on hand OA, (**F**) 1-linoleoylglycerophosphocholine on hand OA, (**G**) X-11423–O-sulfo-L-tyrosine on hand OA, (**H**) 1-linoleoylglycerophosphocholine on finger OA, (**I**) X-11423–O-sulfo-L-tyrosine on thumb OA, (**J**) taurocholate on thumb OA *OA* osteoarthritis

Sensitivity analyses were performed to avoid the horizontal pleiotropy for MR estimate. Figure 3 shows the results of the sensitivity analyses for 10 pairs of metabolites and OA phenotypes with significant causal associations. Generally, the causal association was robust when statistical significances (p < 0.05) were observed in two additional MR tests, typically the Weighted Median test and the MR-PRESSO test. Nine of the 10 pairs of associations were considered to be robust, which respectively were 1-linoleoylglycerophosphocholine on hand OA and finger OA, X-11423–O-sulfo-L-tyrosine on hand OA and thumb OA, isovalerylcarnitine on hand OA, arginine on hip OA, ADpSGEGDFXAEGGGVR* on hip OA, kynurenine on knee-hip OA, taurocholate on thumb OA **(**Fig. 3**)**. The association of 4-acetaminophen sulfate on hip OA showed nonsignificant in the Weighted Median test (p = 0.060), but it still can be considered as a potential causal association as the significance be observed in IVW method and MR-PRESSO test (p = 1.48 × 10^–4^). We further screened out possible horizontal pleiotropy in all associations by MR-Egger’s intercept term and MR-PRESSO's global test (Additional file 1: Table S3). Additionally, scatter **(**Fig. 4**)** and funnel plots **(**Fig. 5**)** ruled out the possibility of potential outliers and horizontal pleiotropy for all identified metabolites. The full results of sensitive and pleiotropy analysis were listed in Additional file 1: Table S4.

### Metabolic pathway analysis

**Fig. 6:**
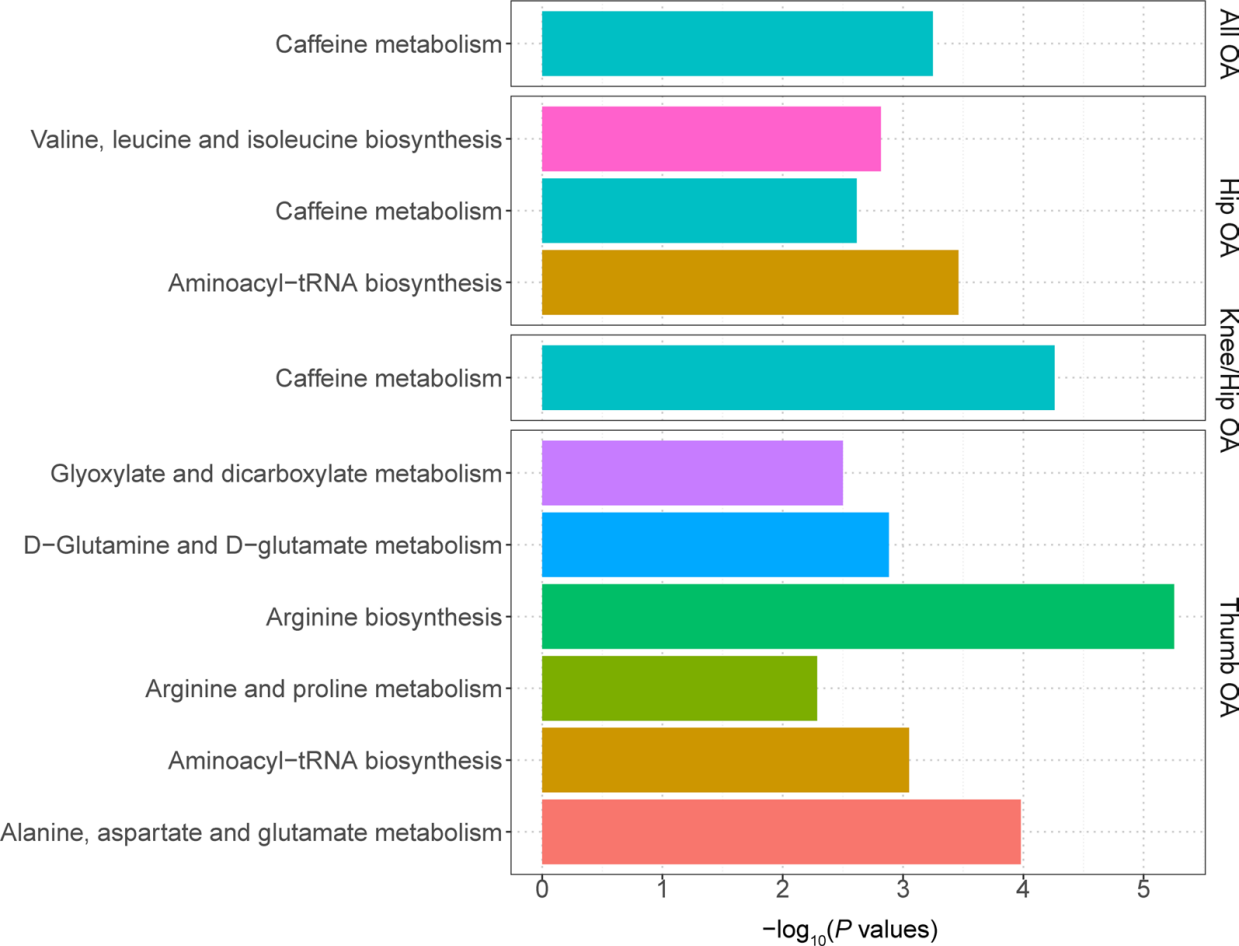
Enriched significant metabolic pathways of 4 OA phenotypes

The metabolic pathway analysis identified 11 significant metabolic pathways in 4 of the included OA phenotypes (Fig. 6). Our results show that the “arginine biosynthesis” (p = 5.58 × 10^–6^), “alanine, aspartate and glutamate metabolism” (p = 1.05 × 10^–4^), “D-Glutamine and D-glutamate metabolism” (p = 0.00131), “glyoxylate and dicarboxylate metabolism” (p = 0.00316) and “arginine and proline metabolism” (p = 0.00518) pathways were found to be associated with the pathogenetic process of thumb OA, whereas “valine, leucine and isoleucine biosynthesis” pathway was considered to be associated with hip OA. We also found some OA phenotypes may share common metabolic pathways, such as the “caffeine metabolism” pathway for all OA (p = 5.65 × 10^–4^), knee-hip OA (p = 5.50 × 10^–5^), and hip OA (p = 0.00243), and “aminoacyl-tRNA biosynthesis” pathway for thumb OA (p = 8.91 × 10^–4^) and hip OA (p = 3.46 × 10^–4^) (Table 2).

**Table 2:** Significant Metabolic Pathways Involved in different OA phenotypes

OA	Metabolite set	Total	Hits	Expect	P value	Holm P	FDR
All OA	Caffeine metabolism	10	2	0.039	5.65E− 04	0.047	**0.047**
Thumb OA	Arginine biosynthesis	14	4	0.137	5.58E− 06	4.69E− 04	**4.69E**− **04**
Thumb OA	Alanine, aspartate and glutamate metabolism	28	4	0.273	1.05E− 04	0.009	**0.004**
Thumb OA	Aminoacyl-tRNA biosynthesis	48	4	0.469	8.91E− 04	0.073	**0.025**
Thumb OA	D-Glutamine and D-glutamate metabolism	6	2	0.059	0.00131	0.106	**0.027**
Thumb OA	Glyoxylate and dicarboxylate metabolism	32	3	0.312	0.00316	0.252	**0.053**
Thumb OA	Arginine and proline metabolism	38	3	0.371	0.00518	0.409	**0.073**
Hip OA	Aminoacyl-tRNA biosynthesis	48	4	0.375	3.46E− 04	0.029	**0.029**
Hip OA	Valine, leucine and isoleucine biosynthesis	8	2	0.063	0.00153	0.127	**0.064**
Hip OA	Caffeine metabolism	10	2	0.078	0.00243	0.200	**0.068**
KneeHip OA	Caffeine metabolism	10	3	0.085	5.50E− 05	0.005	**0.005**

The bold values indicates statistically significant (FDR < 0.05)

## Discussion

**Fig. 7:**
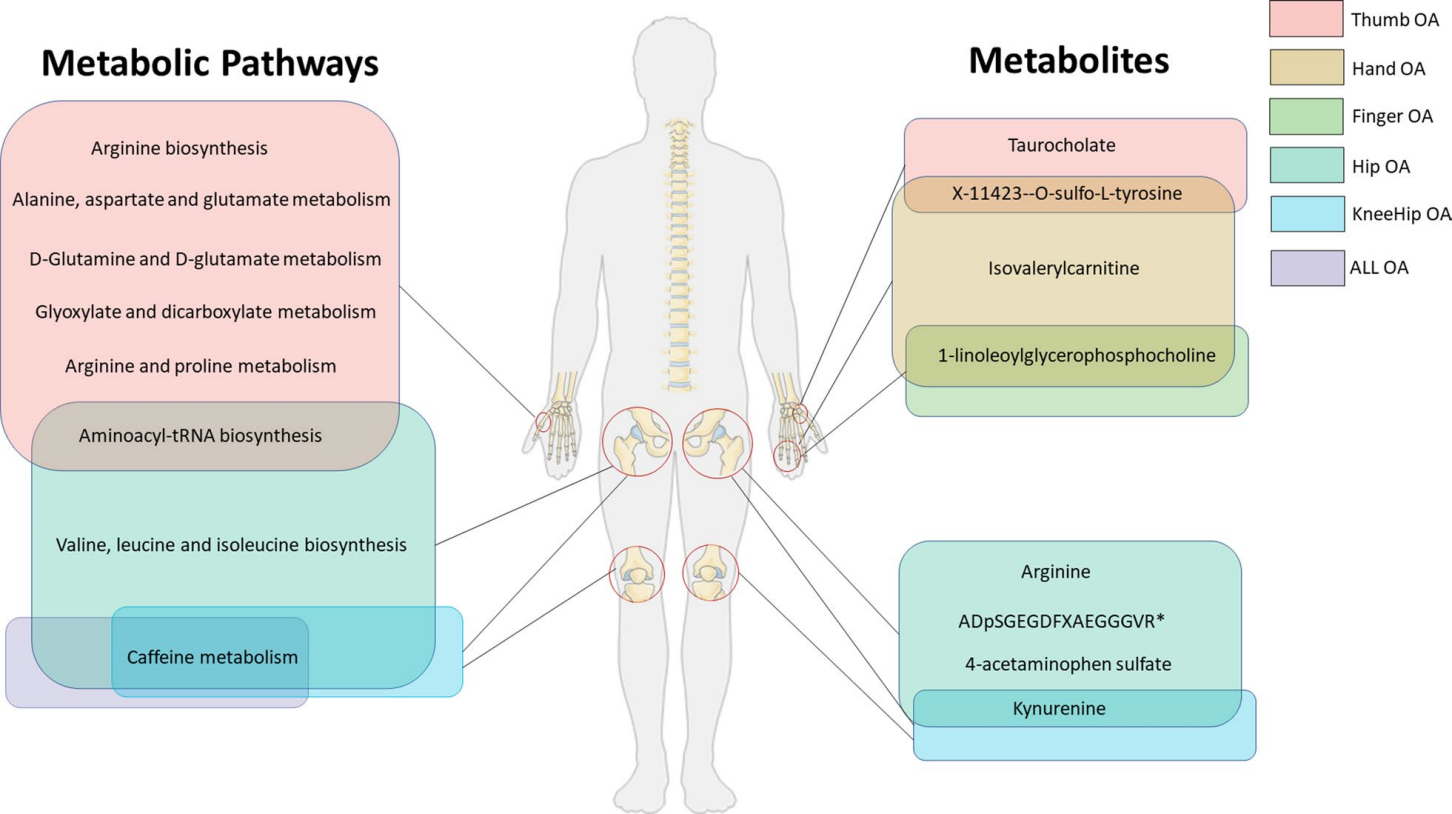
The illustration represents significant causal metabolites and metabolic pathways associated with different OA phenotypes

In this two-sample MR study, we found 235 causal relationships involving 140 metabolites and 8 OA phenotypes, nine of which were observed with multiple-testing corrected significance and considered to be robust associations as they passed all sensitivity analyses. Additionally, eleven significant metabolic pathways involved in 4 OA phenotypes were detected (Fig. 7). To our knowledge, this is the first MR study to systematically appraise the causal role of human blood metabolites in the issue of OA. Our study offers new insights to reveal the role of gene-environment interactions in the pathogenesis of osteoarthritis and to provide potential inspiration for further precision treatments.

In the recent decade, OA has been increasingly recognized as a metabolism-related disease, not only because it can be found in combination with a variety of metabolic disorders, but also because OA-related metabolites and metabolic pathways have been continuously discovered in metabolomic studies [4, 5, 19, 20]. Blood, synovial fluid, cartilage, and subchondral bone are typical sample sources for metabolomic identification [21]. Among them, blood has been considered a good source because it contains numerous detectable metabolites and can easily be obtained in large sample sizes, thus contributing to the screening of circulation markers for OA risk [22, 23]. Metabolomic studies using plasma/serum have identified altered metabolic profiles in patients with OA, with the most commonly reported metabolites being amino acids, taurine, and phospholipids [19, 20]. Our study confirmed the presence of OA-specific metabolic profiles and further identified some key metabolites and metabolic pathways that causally contribute to the pathogenesis of OA.

Our study identified four metabolites from the amino acid metabolism pathways been causally associated with the pathogenic process of OA. Amino acids are not only the components to build peptides and proteins but also precursors for many small molecules (e.g., nitric oxide, dopamine, 5-hydroxytryptamine, polyamines, and glutathione) with irreplaceable physiological functions [24]. Studies have established that amino acids serve as nutrients, immune modulators, and oxidative regulators in joint development and diseases [25]. Among these amino acid-related metabolites in OA, the role of arginine and kynurenine has been increasingly focused on.

Arginine is a semi-essential amino acid involved in the urea cycle and arginine/proline metabolism [26]. In humans, the metabolism of arginine is mainly via the arginase pathway to produce urea and ornithine; and the nitric oxide synthase (NOS) pathway to produce nitric oxide (NO) and citrulline [26]. The anti-inflammatory and antioxidant effects of arginine have been demonstrated in various tissues and cells, and are suggested to act mainly through NO [27]. Existing studies suggested that NO and its redox derivatives may play a protective role in the joints by inhibiting multiple inflammatory pathways and alleviating immune cell infiltrations [27, 28]. Metabolomic analysis indicated the presence of arginine depletion in patients with OA and suggested that it may be due to overactive arginine catabolism [29]. Werdyani et al. [12] identified arginine deficit as one of 3 clinical endotypes of OA, with the other two being muscle weakness and low inflammatory OA. A multi-omic analysis on injury-induced OA animal models also observed an enrichment of arginine metabolism-related genes, suggesting that the arginine metabolic pathway was an important modulated metabolic pathway in OA [9]. Choi et al. [30] observed that the gene encoding Arg-II, an arginine metabolizing enzyme, was specifically upregulated in cartilage samples from both human OA patients and mouse models. They also found that adenovirus-mediated overexpression of Arg-II in mouse joint tissue led to OA pathogenesis and that genetic ablation of Arg2 (Arg2^-/-^) in mice eliminated the pathological manifestations of OA. Li et al. [31] found that exogenous arginine supplementation helped to alleviate lipid peroxidation and inflammatory responses in osteoblasts-osteoarthritis cells, suggesting a novel therapeutic target for OA treatment. Our results further support these findings and highlight the importance of arginine as a protective factor in the progression of hip OA.

As a main degradation product of tryptophan, kynurenine has been shown to have direct damaging effects in a variety of tissues [32–34]. As an essential amino acid for protein synthesis, tryptophan undergoes extensive and complex metabolism along several pathways, resulting in many biologically active molecules acting in different organs through various action mechanisms [35]. Metabolism of tryptophan in the joint mainly involves the kynurenine pathway and the 5-hydroxytryptophan pathway, with the kynurenine pathway being the main pathway in the pathology of OA [36]. Lögters et al. [37] found that kynurenine could inhibit chondrocyte (ATDC5) cell proliferation in a dose-dependent way. In OA rat models, Wang et al. [38] found that activation of the kynurenine-aryl hydrocarbon receptor axis can impair chondrogenesis and chondroprotection in human umbilical cord-derived mesenchymal stromal cells. Based on the given evidence and our findings, kynurenine can be considered an important circulation biomarker and a key therapeutic target for OA.

Carnitine and acylcarnitine are involved in fatty acid β-oxidation and BCAAs (including valine, leucine, and isoleucine) metabolism, and have been demonstrated to have immunomodulatory functions in joints development and diseases [39]. Studies showed that L-carnitine stimulated the proliferation of human primary chondrocytes and may alleviate symptoms of knee pain [40, 41]. Whereas, altered concentrations of multiple acylcarnitines, such as acetylcarnitine, hexanoylcarnitine, and butanoylcarnitine, were observed in OA patients by metabolomic studies [12, 42]. Our MR analysis identified isovalerylcarnitine, another acylcarnitine, to be causally linked to hand OA. Isovalerylcarnitine is a carnitine substrate of the isovaleryl-CoA dehydrogenase, an enzyme that is involved in the degradation of leucine and fatty acids. However, the role of isovalerylcarnitine in OA development has been rarely reported.

Another finding of this study is that we detected 1-linoleoylglycerophosphocholine from the lysolipid pathway had a protective effect on hand OA and finger OA. Dysregulated lipid metabolism has been demonstrated in previous studies to be present in OA as an important pathophysiological feature of the disease [10, 43]. Phospholipids are important components of synovial fluid and help to lubricate joints. Accumulating experimental evidence suggested that disturbed phosphatidylcholine/lysophosphatidylcholine metabolism was a characteristic metabolic alteration in OA and could be used as candidate circulation markers and drug targets [10, 44, 45]. Additionally, phosphatidylcholine/lysophosphatidylcholine metabolism was found to be a shared metabolic pathway between OA and metabolic disorders including diabetes mellitus, partly explaining the concomitant development of OA with these diseases [19, 46]. Known as a lysophosphatidylcholine, although the role of linoleoylglycerophosphocholine in OA has not been reported in the previous literature, studies have confirmed its potential as a biomarker of insulin resistance and impaired glucose metabolism [47]. Given the fact that hand OA, especially the erosive OA subtypes, is closely associated with diabetes [48], 1-lysophatidylcholine could be a promising direction for further studies.

Additionally, we identified X-11423–O-sulfo-L-tyrosine from the phenylalanine and tyrosine metabolism pathway, taurocholate belonging to bile acid metabolism, ADpSGEGDFXAEGGGVR* (a fibrinogen cleavage peptide) and 4-acetaminophen sulfate (a paracetamol related drug metabolite) were causal factors of OA risk. However, mechanisms involved in these metabolites affecting OA have not been fully understood, which warrants further experimental exploration.

In this study, we also identified metabolic pathways that are causally associated with the development of OA, some of which are well documented in experimental studies for their role in the pathogenesis of OA. As mentioned above, arginine-related pathways (including arginine biosynthesis and arginine and proline metabolism) and the valine, leucine and isoleucine biosynthesis pathways have been demonstrated to be involved in the pathogenesis of OA [29, 31]. Glutamine metabolism is involved in biosynthesis and redox reactions. Glutamine metabolism is associated with biosynthesis and redox reactions and has been proven to have a role in regulating cartilage inflammation and cartilage repair [49, 50].

Importantly, our results showed that some OA phenotypes have shared metabolites and pathways. Moreover, there was a visible trend of differential distribution of these shared metabolites among OA in different types of joints. For example, the risky effects of kynurenine were more noticeable in weight-bearing joints including the spine, knee, and hip. Whereas, the causal associations of taurocholate and X-11423–O-sulfo-L-tyrosine with OA were mainly seen in non-weight-bearing joints, such as hand, finger, and thumb. Epidemiological studies have demonstrated differences in the prevalence, risk factors and susceptible populations of OA in weight-bearing and non-weight-bearing joints [1, 51]. Joint type-specific genetic variants have also been observed in GWAS analysis, indicating differences in pathogenic mechanisms between weight-bearing and non-weight-bearing joints [16].

The present study has several strengths. First, the most significant strength of this study is the large scale of genetic variables we covered to analyze the relationship between blood metabolites and different OA phenotypes. Specifically, excluding the ones that have not been identified, a total of 486 metabolites were covered in this study. Meanwhile, the genome-wide data source for the OA genetic variables included 826,690 individuals from 9 populations with 11 different OA phenotypes that can be broadly classified into OA of the weight-bearing joints and non-weight-bearing joints. Benefiting from these two GWAS datasets, we have made our study a relatively comprehensive and systematic analysis of the metabolic profile related to OA development. Second, using the MR design, our study largely avoided reverse causality and residual confounding factors. As extensive sensitivity analyses ruled out the possibility of variable polymorphisms, inferences on causal relationships between metabolites and OA risk in this study were considered to be robust.

There are still some limitations in our study. Firstly, this MR study was a blood metabolome-based analysis. Although blood has been suggested as a good source of sample for metabolite data, as the direct pathological site of osteoarthritis is within the joint cavity, further studies to analyze metabolite changes in synovial fluid or cartilage would help identify more promising biomarkers and drug targets. Secondly, the data for metabolites were mainly from European populations, which limited the cross-ethnic extrapolation of our findings. Thirdly, this study covered a relatively comprehensive spectrum of metabolites, however, the functions and mechanisms of some metabolites in the disease were not fully understood, which limited our interpretation of our findings from this MR analysis.

## Conclusion

In conclusion, this is the first systematic MR analysis to evaluate the causal relationship between serum metabolites and different OA phenotypes using genome-wide data, providing preliminary evidence on the impact of circulating metabolic disturbance on the risk of OA. By IVW and multiple sensitive analysis, nine robust causal associations between 7 metabolites and 5 OA phenotypes were finally identified. Eleven significant metabolic pathways in 4 OA phenotypes were identified by metabolic pathway analysis. The finding of our study suggested that these metabolites can consider useful circulating metabolic biomarkers for OA screening and prevention in clinical practice, and can also serve as candidate molecules for future mechanism exploration and drug target selection.

## Supplementary Information

**Additional file 1** **Table S1**: Information of the instrumental variables an SNPs identified by Mendelian randomization estimates. **Table S2**: Raw data of the results from IVW analysis between metabolites and 8 osteoarthritis phenotypes. **Table S3**: The results of MR analysis for 10 causal associations with multiple test correction significance. **Table S4**: Raw data of the results of sensitivity analysis between all metabolites and osteoarthritis phenotypes. **Table S5**: Raw data of the results for the metabolic pathway analysis.

## Data Availability

Publicly available datasets were analyzed in this study. This data can be found here: http://metabolomics.helmholtz-muenchen.de/gwas/. All the data generated by the MR analysis is in the included Supplementary Material.
